# Adhesion Process of Biomimetic Myelin Membranes Triggered by Myelin Basic Protein

**DOI:** 10.3389/fchem.2021.631277

**Published:** 2021-05-04

**Authors:** Benjamin Krugmann, Alexandros Koutsioubas, Luman Haris, Samantha Micciulla, Didier Lairez, Aurel Radulescu, Stephan Förster, Andreas M. Stadler

**Affiliations:** ^1^Jülich Centre for Neutron Science at MLZ, Forschungszentrum Jülich GmbH, Garching, Germany; ^2^Institute of Physical Chemistry, RWTH Aachen University, Aachen, Germany; ^3^Jülich Centre for Neutron Science (JCNS-1) and Institute for Biological Information Processing (IBI-8), Forschungszentrum Jülich GmbH, Jülich, Germany; ^4^Institut Laue-Langevin, Grenoble, France; ^5^Laboratoire des Solides Irradiés, École Polytechnique, CEA, CNRS, Institut Polytechnique de Paris, Palaiseau, France

**Keywords:** neutron reflectometry, adhesion energy, lipid membranes, myelin basic protein, vesicle fusion, random sequential adsorption, myelin sheath

## Abstract

The myelin sheath—a multi-double-bilayer membrane wrapped around axons—is an essential part of the nervous system which enables rapid signal conduction. Damage of this complex membrane system results in demyelinating diseases such as multiple sclerosis (MS). The process in which myelin is generated *in vivo* is called myelination. In our study, we investigated the adhesion process of large unilamellar vesicles with a supported membrane bilayer that was coated with myelin basic protein (MBP) using time-resolved neutron reflectometry. Our aim was to mimic and to study the myelination process of membrane systems having either a lipid-composition resembling that of native myelin or that of the standard animal model for experimental autoimmune encephalomyelitis (EAE) which represents MS-like conditions. We were able to measure the kinetics of the partial formation of a double bilayer in those systems and to characterize the scattering length density profiles of the initial and final states of the membrane. The kinetics could be modeled using a random sequential adsorption simulation. By using a free energy minimization method, we were able to calculate the shape of the adhered vesicles and to determine the adhesion energy per MBP. For the native membrane the resulting adhesion energy per MBP is larger than that of the EAE modified membrane type. Our observations might help in understanding myelination and especially remyelination—a process in which damaged myelin is repaired—which is a promising candidate for treatment of the still mostly incurable demyelinating diseases such as MS.

## Introduction

The biological membrane is an important component of cellular function and metabolism. Investigation of biological membrane components, characterization of their physico-chemical properties and the study of their interactions with membrane binding proteins can answer important questions that are central for human health and disease. Several human diseases are directly connected to modification of cellular membranes (Evans, 1980; Maret et al., 1983; Vignini et al., 2007). Degeneration of the well-ordered myelin sheath that exists around nerve cells in the human brain, for instance, results in nerve conduction failure and neurodegeneration (Love, 2006; Weil et al., 2016). This phenomenon is called demyelination. Myelin basic protein (MBP) stabilizes the myelin membrane multi-layer and is an integral part of the myelin sheath (Boggs, 2006). Of particular relevance is the interaction of MBP with cytoplasmic leaflets of oligodendrocytes. Those cells are assembled to each other by MBP to generate a double bilayer membrane unit which envelopes the axons as a multi-membrane stack forming compact myelin (Quarles et al., 2006). Multiple sclerosis (MS) is a chronic inflammatory disease of the central nervous system, correlated with demyelination through membrane de-adhesion, swelling, and ultimately vesiculation of the myelin sheath (Weil et al., 2016). The lipid compositions of native (healthy) and modified (diseased) myelin sheaths have been investigated in an animal model (Ohler et al., 2004). While the native lipid composition occurs in healthy cytoplasmic myelin sheaths, the diseased lipid composition has been found in animals having experimental autoimmune encephalomyelitis (EAE), which is an animal model to study diseases such as e.g., MS that are connected to demyelination (Ohler et al., 2004). Structural properties of biomimetic native-like and EAE-diseased membranes have been investigated *in vitro* in a biosynthetic approach as oriented membrane bilayers or in the vesicle state (Min et al., 2009, 2011; Shaharabani et al., 2016, 2018; Raasakka et al., 2017). Recently, we found a direct connection between the bending stiffness of large unilamellar vesicles (LUV) having either EAE-diseased or native-like lipid composition and the formation of multilamellar structure which is mediated by the binding strength of MBP with the respective membrane types (Krugmann et al., 2020b).

While most of the above-mentioned studies have investigated structural aspects of those biomimetic myelin-like model membranes in their equilibrium state, knowledge about molecular properties that regulate the kinetics of the assembly process of the myelin sheath is currently still largely missing. A detailed molecular understanding of the formation of the myelin membrane systems is required both from a fundamental biological and biophysical point-of-view as well as to develop novel approaches for medical treatment of neurodegenerative diseases using a process known as remyelination. Remyelination is a process during which a degenerated myelin membrane is replaced by a new sheath, which seems to be thinner than the original, but it is still capable to maintain biological function of myelin (Prineas and Connell, 1979; Franklin and Ffrench-Constant, 2008). There are high expectations to utilize this natural process for the treatment of human demyelinating disorders such as, for instance, MS. Therefore, many efforts are made to understand the molecular mechanism of this process in detail. Currently, knowledge regarding the details of the molecular process of remyelination using vesicle fusion is still missing.

In our study, we investigated molecular aspects of the myelin formation process by using biomimetic membrane systems. We first produced a supported membrane bilayer (SMB) mimicking native or diseased-like cytoplasmic myelin on a silicon substrate and coated it with a dense MBP layer on top. The adsorption kinetics of LUV having the same lipid composition as those bilayers were then studied using neutron reflectometry (NR) as a function of incubation time. NR experiments performed under steady-state conditions at different stages of the assembly process allowed us to gain structural information of the oriented membrane systems orthogonal to the membrane plane with resolution on the nm length-scale. The kinetic adhesion mechanism of the LUV could be modeled by a random sequential adsorption (RSA) process. As the maximal adsorption area of RSA process is known as well as the bending rigidity of the LUV membrane, we could calculate the interaction energy per MBP protein. This parameter was found to be larger for native lipid composition as compared to the EAE-diseased membranes. A perspective for a future application of our approach would be to form myelin-like multilayers by LUV fusion, which would be an interesting mechanism to externally trigger remyelination by LUV fusion.

## Materials and Methods

### Materials

Porcine brain lipids (PC, PE, PS, and SM) and ovine cholesterol were purchased from Avanti Polar Lipids (Alabaster, AL, USA) and bovine MBP from Sigma-Aldrich (St. Louis, MO, USA).

### Liposome Preparation

Porcine brain lipids (PC, PE, PS, and SM) with chain compositions given on the Avanti webpage^1^ and ovine cholesterol have been separately dissolved in chloroform, or bought in chloroform. The lipids are mixed in ratios found in native and EAE modified myelin (Table 1; Krugmann et al., 2020b) and afterwards the chloroform is evaporated using a gentle nitrogen stream followed by vacuum annealing at 50°C overnight. The lipids are then dissolved in D_2_O-buffer [150 mM NaCl, 10 mM 3-(N-morpholino) propanesulfonic acid (MOPS)]. The solution is shaken until the lipid cake is no longer stuck at the glass surface. If necessary remaining lipid cake is removed by pipetting. In the following the solution is sonicated for 30 min at 40°C and freeze-thawed 5 consecutive times. To remove remaining large aggregates or giant vesicles the solution is centrifuge-filtered through a 0.45 μm for 10 min at 10,000 g-force. Finally, the solution is extruded through a 100 nm membrane 21 × at 50°C.

**Table 1:** Lipid compositions of native and EAE modified cytoplasmic myelin membranes (Krugmann et al., 2020b).

**Lipid type**	**Native (mol %)**	**EAE modified (mol %)**
PC	25.9	20.1
PE	29.0	32.9
PS	7.0	7.4
SM	6.2	2.2
Cholesterol	31.6	37.4

### Supported Bilayer Preparation

Polished silicon blocks were cleaned by subsequent immersion in chloroform, acetone, ethanol and water under sonication for 20 min each, followed by UV/ozone irradiation for 30 min and rinsing in H_2_O to verify hydrophilicity. All silicon substrates were stored in water until utilization to prevent contamination. For the neutron reflectometry measurements, each substrate was sealed in a solid/liquid cell. In detail, the bottom part of the cell is equipped with a PEEK through, which represents the liquid reservoir, while the upper part is an aluminum/Teflon lid. The silicon block is sandwiched between the two parts and the trough (reservoir) filled with Milli-Q water by injection through Teflon tubes (0.5 mm inner diameter), closed by valves. Prior to liposomes solution injection, the cell was pre-heated at 50°C to promote vesicle fusion. When the liposomes were in contact with the hydrophilic SiO surface, a supported membrane bilayer (SMB) was formed and residual liposomes were removed by rinsing with buffer solution.

### NR Protocol

Neutron reflectivity measurements were performed on the neutron reflectometer D17 at the Institut Laue-Langevin (ILL, Grenoble, France) (Saerbeck et al., 2018; Krugmann et al., 2020a) operated in time-of-flight mode. The instrumental resolution ΔQ/Q was varying between 2 and 10% along the full *q*-range (0.006 0.35 *A*^−1^) with accessible wavelengths from 2 to 30 Å and two angles of incidence (0.8 and 3.2°).

The footprint length (in the beam direction) and width (in the perpendicular direction) were *L* = 60 mm and *W* = 35 mm, respectively.

The samples were deposited at the solid/liquid interface of a silicon substrate sealed inside a solid/liquid cells with 1 mL liquid reservoir which was kept at 50°C to promote vesicle fusion; during the rest of the experiment a constant temperature of 37°C was maintained via a thermostatic bath. Contrast variation was carried out via an automatized pumping system at a flow rate of 1 mL/min for a full volume exchange of 20 mL. Firstly, the silicon blocks are characterized in three contrasts (D_2_O-buffer, H_2_O-buffer, silicon matched water (SMW)-buffer (38% D_2_O). Afterwards the respective SMB is formed using the protocol described above. Now, the SMBs are characterized in the same three contrasts. We inject MBP in the cells to coat the SMB as described in Krugmann et al. (2020b). To remove remaining MBP solution the cells are flushed. Again, the membrane is measured at the three contrasts. Finally, the vesicles are injected and the adsorption kinetics is monitored (in D_2_O). To enable very fast acquisition the beam divergence is increased and a time resolution of 1 min can be achieved (Cubitt et al., 2015).

### NR Data Analysis

For the specular NR, the data first has been reduced using the COSMOS tool in the LAMP software of the ILL. The fitting of those reduced datasets was done using the Anaklasis package based on python (Koutsioubas, 2021). This software allows co-refining multiple datasets as e.g., the same sample at different contrasts or the same sample in D_2_O at different steps of the kinetic process. The model assumed is a stratified layer model, while an additional adjustable multiplicative parameter is used for the proper scaling of the reflectivity curves. During data reduction a normalization factor is set accounting for neutron beam attenuation as defined by a direct beam measurement. In the pristine membrane samples only a small deviation to unity is observed which could be explained by a not perfect sample alignment. In the later steps the deviation from unity increases especially in the samples with attached vesicles. This might be accounted to diffuse scattering in these samples. Therefore, less signal is scattered specular and a value smaller than unity needs to be chosen as scaling factor. For the bare membrane we assume the layer order head|chain|head|. This layer sequence will just be called membrane in the following. When adding MBP an additional layer is added and the order is changed to membrane|MBP. After injection of the vesicles the order is changed to membrane|MBP|membrane|diffuse vesicle layer. The diffuse vesicle layer accounts for the 3D structure of the attached vesicles and has a SLD of 0.7 · 10^−6^ Å^−2^ between the one of the chain and the head section. As front end we use in all cases Si|SiO|buffer and as back end buffer. Obtained membrane thicknesses are compiled in Table 2.

**Table 2:** Coverage of the second membrane bilayer η, thicknesses of the membrane parts, MBP concentrations in the protein layers, and MBP layer thickness *d*_MBP_.

	**Native01**	**Modified01**	**Modified1**
c*_MBP_ (mg/ml)	0.1	0.1	1
η	0.26 ± 0.02	0.34 ± 0.02	0.39 ± 0.02
*d*_head_ (Å) (inner)	7.9± 1.6	11.1 ± 2.0	8.1± 1.4
(outer)	9.8± 1.5	11.5 ± 1.7	11.3 ± 1.3
*d*_chain_ (Å)	31.2 ± 0.8	32.6 ± 0.8	31.6 ± 0.7
*d*_MBP,SBL_ (Å)	77 ± 68	64 ± 44	72 ± 20
*d*_MBP,DBL_ (Å)	30.6 ± 2.0	26.4 ± 1.6	24.6 ± 1.3
*c*_MBP,SBL_ (vol. %)	3 ± 2	5 ± 3	7 ± 2
*c*_MBP,DBL_ (vol. %)	1 ± 3	0 ± 4	11 ± 3

* *MBP concentration in the injected buffer solution. The errors of the fitted parameters are taken from the fit*.

### Random Sequential Adsorption Simulation

The RSA simulation is modified from Erban and Chapman (2007) for our system. As starting point we take a test volume (*x*-*y*-*z* = 1 μm – 1 μm – 1 mm) with a constant concentration of vesicles in *z*-direction. In practice a test area (*x*-*y*) is defined. The *x*-*y*-surface at *z* = 0 is the adhesive surface. Then we define vesicles in *z*-direction. Since the concentration should be uniform in *x*-*y*-direction we only have to calculate the diffusion of vesicles in *z*-direction and only initialize the *x*-*y*-position in the case that the vesicles touch the surface.

Now we start the diffusion simulation. Therefore, we calculate the diffusion of each vesicle in time steps Δ*t* by

(1) $$\begin{array}{l} z_{new}=z_{old}+\sqrt{2D\Delta t}\cdot \chi \end{array}$$

Here, *D* is the diffusion constant and χ is a normal distributed random number.

In the case that the vesicles diffuse to the adhesive surface and no other adhered vesicle is overlapped in *x*-*y*-position they get adsorbed by a certain probability and are removed from the simulation. This is done in the simulation in the case that one of two conditions are fulfilled. The first condition is that the end *z*-position is negative. If this condition is fulfilled the vesicle adsorbs with the probability:

(2) $$\begin{array}{l} p=P\cdot \sqrt{\Delta t} \end{array}$$

In Erban and Chapman (2007) *P* describes a positive constant which can be related to the rate constant of the chemical reaction between the virus surface and diffusing polymers. As the geometry of our system is similar in our case *P* is related to the interaction of vesicles and the MBP coated membrane surface. The second condition checks if the vesicles *z*-position was negative during the step. The adsorption probability is given by:

(3) $$\begin{array}{l} p=\exp\left(-z_{old}\frac{z_{new}}{D\Delta t}\right)\cdot P\cdot \sqrt{\Delta t} \end{array}$$

At the beginning only, the vesicle concentration close to the surface gets perturbed by the surface adhesion. Therefore, only vesicle diffusion close to the surface is calculated in the beginning. The affected *z*-range *L*(*t*) increases is given by:

(4) $$\begin{array}{l} L\left(t\right)=2\cdot {\text{erf}}^{-1}0.99\cdot \sqrt{tD} \end{array}$$

In this model we assumed the vesicles with diameter *d* = 100 ± 30 nm as spherical hard shells which exclude the area $A_{ex}={\frac{\pi }{4}d}^{2}$ when attached to the membrane. The simulation has been run 10 consecutive times and the results have been averaged. The time step was set to Δ*t* = 1 s. We estimated a value $P\;=\;0.015\cdot 1/\sqrt{t}$ leading to a quite convincing agreement with the measured data. The maximum coverage of such a system has been determined before (Wang, 1994; Cieśla and Nowak, 2016; Cieśla and Ziff, 2018) to be 55.47% for monodisperse disks—which is a good description of the 2D-projection of the vesicles on the membrane surface. In case of polydispersity this value increases as vesicles with smaller diameter can fit in smaller holes (Adamczyk et al., 1997). We simulated the effect for a polydispersity of 0.3 (Supplementary Figure 1) which we measured for vesicles of the same compositions and almost identical extrusion procedure via SANS and SAXS (Krugmann et al., 2020b). The coverage seems to be almost completely stable over time—at a time range relevant for the incubation time of the vesicles until the static measurements were started (native ~20 h, EAE modified ~10 h). After 1 day we simulated a steric vesicle coverage of 0.60 ± 0.05 (the error was estimated from the incubation time range of the samples) which is used in the further calculations as explained in the following chapter. Here, we used a time step Δ*t* = 10 s in the simulation. The concentration of vesicles was chosen to be 1 mg/ml. From this value the number concentration was calculated by estimating the weight of one vesicle.

### Accessing the Adhesion Constant *k*_a_

#### Free Energy of a Vesicle

Let us consider a vesicle of area *A*, supposed to be constant upon deformation, adsorbed onto a plane. Its free energy reduces to two terms:

(5) $$E_{free}=E_{a}+E_{b}\;with\;\{\begin{array}{c} E_{a}=-k_{a}\cdot \alpha_{f}A\\ E_{b}=\frac{\kappa }{2}\int {\left(c_{1}\left(s\right)+c_{2}\left(s\right)\right)}^{2}ds \end{array}$$

The first term *E*_*a*_ is the adhesion energy potential, with *k*_a_ the adhesion constant and α_*f*_ the area-fraction of the vesicle surface that is flat and adheres to the plane. This term decreases with increasing adhesive-area fraction α_*f*_.

The second term *E*_*b*_ is the bending elastic potential that can be viewed as the variance of the local curvatures *c*_1_(*s*) and *c*_2_(*s*) of the surface element *ds*, with κ the mean-curvature modulus of the bilayer. This term increases as the shape deviates from the sphere, i.e., for increasing flat part given by α_*f*_.

For a given set (α_*f*_, *k*_*a*_, κ), the vesicle adopts the shape with local curvatures (*c*_1_(*s*), *c*_2_(*s*)) allowing $\int {\left(c_{1}\left(s\right)+\;c_{2}\left(s\right)\right)}^{2}ds$ to be minimized with the constraint of curvature-continuity at the boundary of the flat area. Since this constraint depends only on α_*f*_, so does the least-energy shape of the vesicle. Determining this least-energy surface is the first step for the calculation of *E*_*free*_. For this we followed the procedure described in Koutsioubas et al. (2017). The fundamental assumption is the following: the least- energy surface of an adsorbed vesicle is a solid of revolution with a generatrix that is itself a least-energy curve. The main advantage of this method is to reduce the problem to a 1D-problem that can be exactly solved with a minimum computing-time consumption. Actually, the mean-curvature modulus *k*_c_ can be independently measured or kept from the literature. So that, *E*_*free*_ can be finally computed in the plane (*k*_*a*_, α_*f*_) (see Supplementary Figure 2). For a given adhesion constant *k*_a_, vesicles adopt the value for the adhesive-area fraction α_*f*_ allowing *E*_*free*_ to be minimized, i.e., in the valley of Supplementary Figure 2. Conversely, by measuring α_*f*_ one can determine the corresponding value for *k*_*a*_. The bending rigidity κ of native and EAE-diseased membranes was determined by NSE previously (Krugmann et al., 2020b) and the values of κ are compiled in Table 3. Obtained values of α_*f*_ and *k*_*a*_ determined in this study are summarized in Table 3 as well.

**Table 3:** Values of the bending rigidity κ, the membrane coverage η, the fraction of the vesicles which are flat α_*f*_, the calculated adhesion energy *k*_*a*_, the area per MBP molecule *A*_*MBP*_ and the adhesion energy per MBP molecule ϵ_*MBP*_.

	**Native01**	**Modified01**
κ (10^−19^*J*)^#^	1.44 ± 0.10	1.16 ± 0.06
η	0.26 ± 0.02	0.34 ± 0.02
α_*f*_	0.138 ± 0.022	0.198 ± 0.028
$k_{a}\;\left(mJ/m^{2}\right)$	0.36± 0.06	0.45 ± 0.10
*A*_*MBP*_ (*nm*^2^)^$^	156 ± 172	113 ± 103
*A*_*MBP*_ (*nm*^2^)^#^	86 ± 17	50 ± 15
$\epsilon_{MBP}\;\left(10^{-19}J\right)$	0.31 ± 0.08	0.23 ± 0.08

# *Values of the bending rigidity and area per MBP are taken from Krugmann et al. (2020b).*

$ *Values of area per MBP calculated from fit results in Table 2*.

#### Combining RSA Simulations and Neutron Reflectivity Measurements to Access α_f_

“From the top” point of view (RSA):

In the RSA model, the addition of a new particle is sensitive to the steric hindrance of particles already adsorbed. Let us denote *A*_*ex*_ the maximum extension area parallel to the plane of a vesicle, i.e., its projection area on the plane, and define the “steric coverage density” ρ as:

(6) $$\begin{array}{l} \rho =N\cdot \frac{A_{ex}}{A_{tot}} \end{array}$$

where *N* is the number of adsorbed vesicles and *A*_*tot*_ the total area of the plane.

“From the bottom” point of view (neutron reflectivity):

On the other side, neutron reflectivity is sensitive to the area of vesicle that adheres and is directly in contact with the plane. Let us denote *A*_*f*_ this area with no curvature per vesicle and define the “adhesive coverage density” η as:

(7) $$\begin{array}{l} \eta =N\cdot \frac{A_{f}}{A_{tot}} \end{array}$$

Dividing Equation (6) by Equation (7) gives:

(8) $$\begin{array}{l} \frac{\rho }{\eta }=A_{ex}/A_{f} \end{array}$$

By definition, *A*_*f*_ writes:

(9) $$\begin{array}{l} A_{f}=\alpha_{f}A \end{array}$$

But actually, the flatter the vesicle, the wider it is, so that *A*_*ex*_ also depends on α_*f*_. For a spherical vesicle of radius *R*, one has $A_{ex,a_{f}\;=\;0}=\;\pi R^{2}$ and for a completely flattened vesicle $A_{ex,a_{f}\;=\;0.5}\;=\;A/2\;=\;2\pi R^{2}\;=\;2A_{ex,\alpha_{f\;}=\;0}$. The computation of the least-energy surface as a function of α_*f*_ shows that the departure from the linearity between these two extremities is negligible (see Supplementary Figure 3).

This leads to

(10) $$\begin{array}{l} A_{ex}≃A\cdot \left(1+2\alpha_{f}\right)/4 \end{array}$$

Equations (8) and (10) give: ρ/η = (1 + 2α_*f*_)/4α_*f*_ leading to

(11) $$\begin{array}{l} \alpha_{f}≃\frac{1}{4\left(\frac{\rho }{\eta }\right)-2} \end{array}$$

Considering the situation at time t → ∞ with ρ_∞_ = 0.60 ± 0.05 is estimated from the simulations, one can deduce α_f_ from the neutron reflectivity measurement of η_∞_.

## Results and Discussion

**Figure 1:**
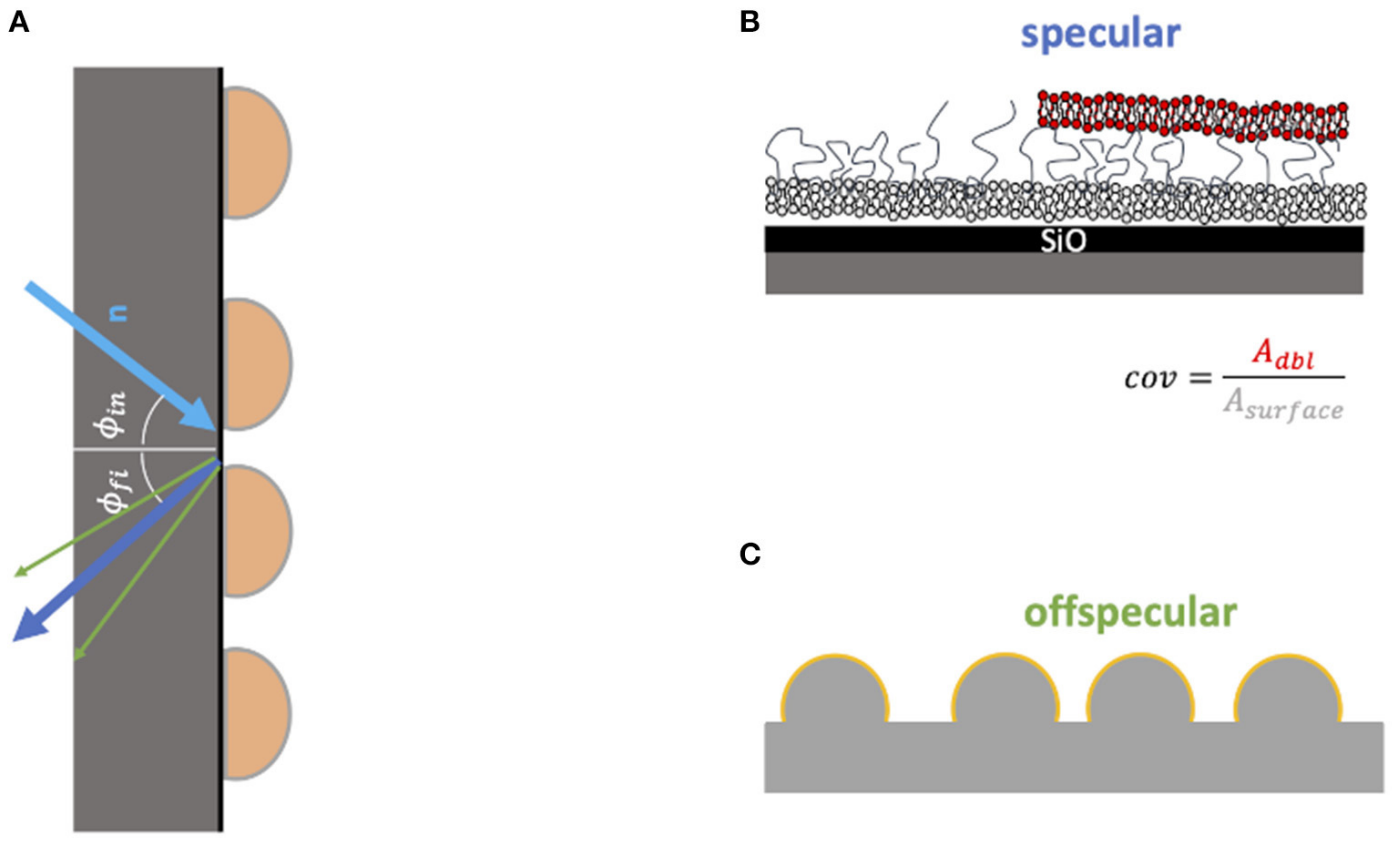
Model of structures seen by the specular **(A,B)** and off-specular **(C)** NR signal. In the specular signal the layer structure in *z* direction is measured very precisely. However, it is more sensitive for the structure close to the silicon surface. From the fit of the specular data the coverage of the second bilayer can be estimated (top right panel). In the off-specular signal the roughness of the layer is measured **(C)**. If this signal appears one can conclude that the layer structure is not flat.

To precisely analyze the interaction of MBP coated membranes with other membranes we choose to rely on neutron reflectometry (NR). Information is obtained perpendicular to the membrane plane by detection of the specular NR signal in combination with in-plane resolution by also measuring off-specular NR data (Zhou and Chen, 1995; Jablin et al., 2011). This is shown schematically in Figure 1. The reflected neutron beam is mostly scattered specular—meaning that the final angle Φ_*fi*_ is equal to the initial angle Φ_*in*_. The specular signal is determined by the structure perpendicular to the surface and has a resolution in the order of a few Ångstrom. However, if there is also in plane structure in the sample—in most cases roughness—a part of the beam is reflected at a different angle. This fraction is called off-specular and is only sensitive to parts of the sample which have in-plane structure.

### Preparation of the Membrane System and Steady-State Characterization

**Figure 2:**
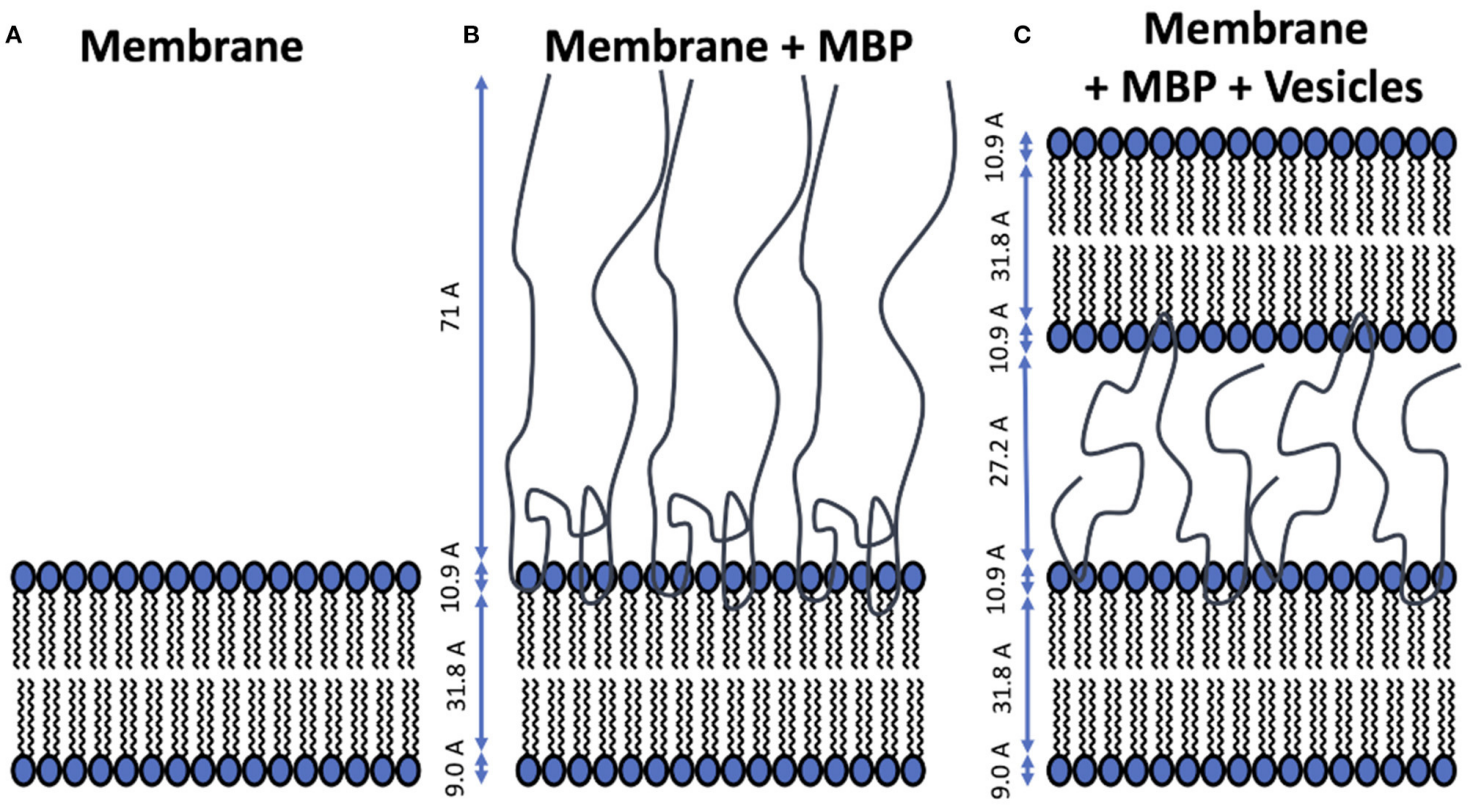
Schematic representation of structural models for the different stages of the experiment. The averaged thickness values of the membrane components are indicated in the figure panels. **(A)** Supported membrane bilayer formed by vesicle fusion on silicon wafer. **(B)** MBP-coated membrane formed after injection of protein solution and incubation. **(C)** Double-layer membrane structure connected by a dense MBP phase in between that is formed upon adhesion of the LUVs.

The system of interest is a supported membrane bilayer (SMB) coated with MBP which is kept in a liquid cell. Into this cell large unilamellar vesicles (LUV) are injected which can diffuse toward the SMB surface and adhere to it. The main purpose of our study was to investigate the mechanism of that adhesion process. Prior to that we validated the structural properties of the membrane components—an essential prerequisite for the interpretation of the kinetic NR experiments. The different stages of the preparation process are schematically depicted in Figure 2 alongside with the average thicknesses of the membrane components.

**Figure 3:**
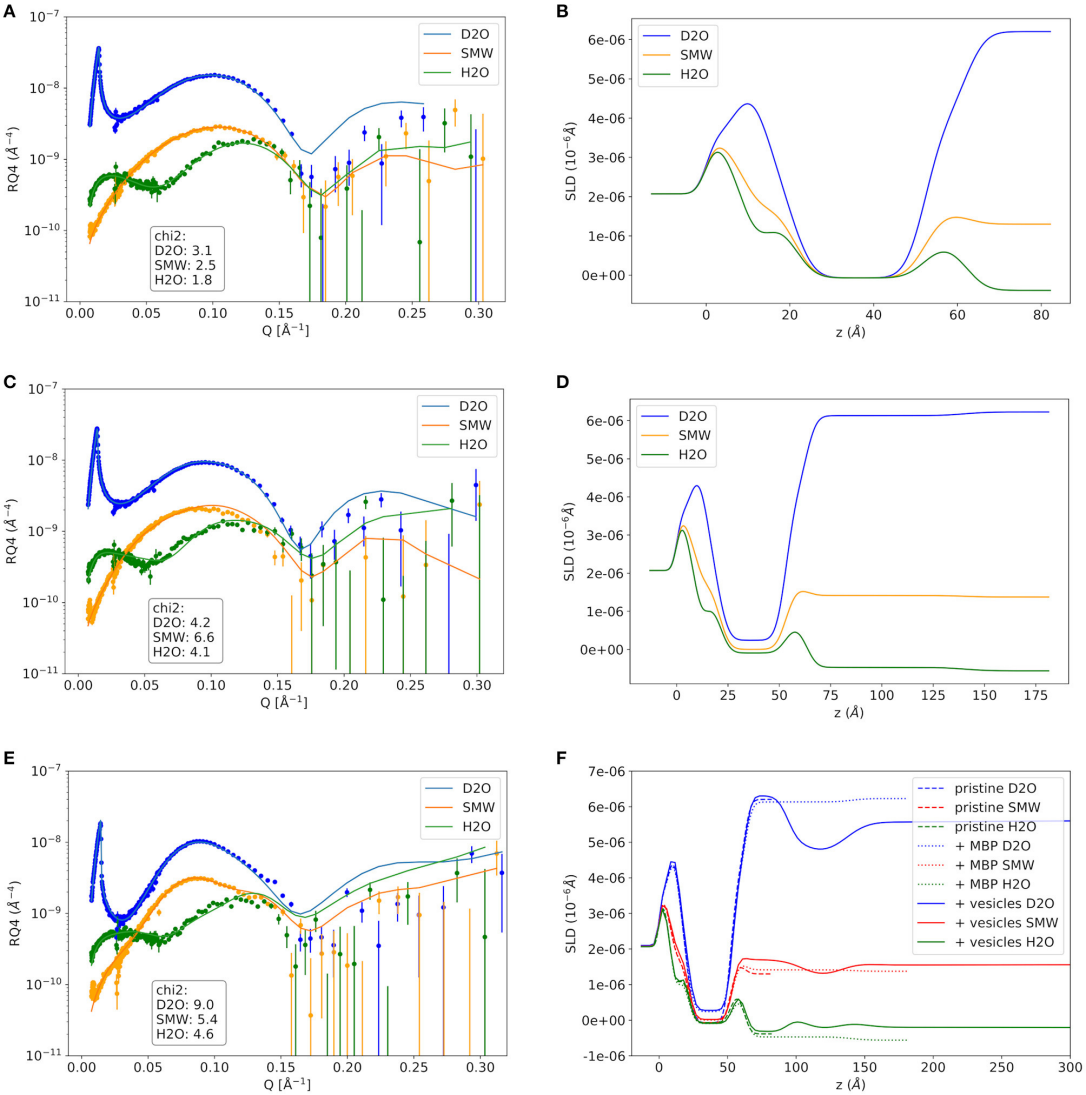
Neutron reflectivity curves and respective SLD profiles of the Native01 sample. **(A,B)** The pure native membrane without MBP, **(C,D)** the supported membrane with MBP protein layer on top that was formed by addition of protein solution with 0.1 mg/ml MBP, and the double-membrane structure with dense intramembrane MBP-layer that was formed after vesicle adhesion in **(E,F)**. In **(F)** the SLD profiles of the pristine membrane and the MBP coated membrane are shown as comparison. NR data was measured on the D17 neutron reflectometer.

**Figure 4:**
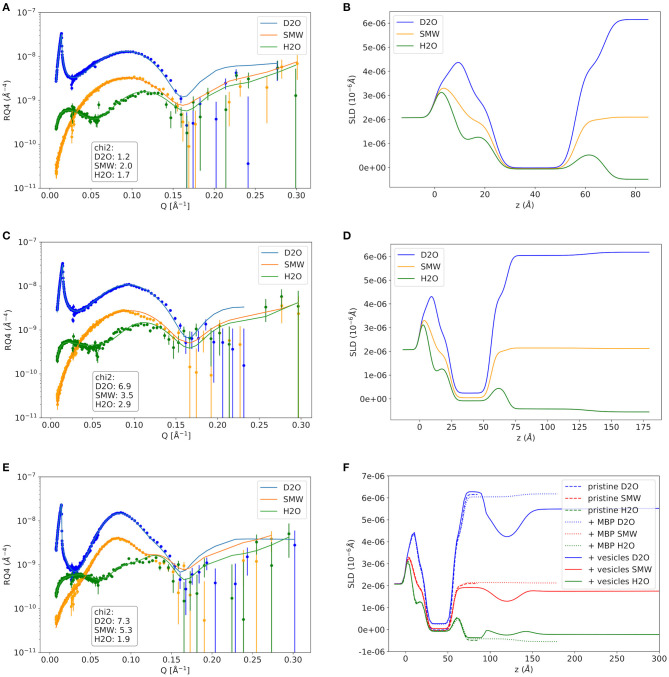
Neutron reflectivity curves and respective SLD profiles of the EAE Modified01 sample. **(A,B)** The pure EAE membrane without MBP, **(C,D)** the supported EAE membrane with MBP protein layer on top that was formed by addition of protein solution with 0.1 mg/ml MBP, and the double-EAE-membrane structure with dense intramembrane MBP-layer that was formed after vesicle adhesion in **(E,F)**. In **(F)** the SLD profiles of the pristine membrane and the MBP coated membrane are shown as comparison. NR data was measured on the D17 neutron reflectometer.

As a first step the SMB was created by vesicle fusion on a hydrophilic silicon substrate (Figure 2A; Stroumpoulis et al., 2006; Rondelli et al., 2017). The neutron reflectometry curves were measured on the neutron reflectometer D17 at the ILL (Saerbeck et al., 2018) at three different contrasts having buffer compositions of 100% D_2_O, 100% H_2_O and a mixture (38%/62% v/v) D_2_O/H_2_O which matches the SLD of silicon being abbreviated as silicon matched water (SMW) in the following. The NR data are shown for a native and EAE modified SMB in Figures 3A, 4A, respectively. The fit of a simple head—chain—head layer sequence can reproduce the measured NR data nicely (see Figures 3A, 4A). A SMB with native-like lipid composition and two SMBs with EAE modified lipid composition referred to as Native01, Modified01 and Modified1 SMBs were characterized. The obtained thicknesses of the head (*d*_head_) and chain sections (*d*_chain_) of those three SMBs are reported in Table 2 and the respective water penetration values are given in Supplementary Table 1. The respective SLD profiles are shown in Figure 3B of the Native01 membrane and in Figure 4B of the Modified01 SMB.

As a next step MBP was injected in the liquid cell at either a concentration of *c*_MBP_ = 0.1 mg/ml for the Native01 and Modified01 SMBs or at 1 mg/ml for the Modified1 SMB. Due to electrostatic interaction, it interacts with the negatively charged membrane and forms a dense protein layer on top of the membrane (see Figure 2B). The data of the Native01 membrane with 0.1 mg/ml MBP in combination with a fit is shown in Figure 3C. The respective data of EAE modified membrane Modified01 with 0.1 mg/ml MBP including theoretical fit is shown in Figure 4C. Those datasets have been fitted as a layer sequence head—chain—head and an additional dense MBP layer on top of the membrane with thickness *d*_MBP,SBL_ and concentration *c*_MBP,SBL_. During these fits the fitted values of *d*_head_ and *d*_chain_, water penetration and roughness between the layers from the SMB without MBP were used and constrained in ±5% of the best value around those values, while the thickness of the MBP layer *d*_MBP,SBL_ (constrained for Modified01: 60–100 Å, otherwise: 70–100 Å) and its water penetration were fitted. The water penetration of the chain section was left free between 0 and 5%. The SLD of MBP was estimated with the primary structure of bovine MBP using the Biomolecular Scattering Length Density Calculator^2^ considering 90% exchange of labile hydrogen atoms. These values are in D_2_O $\rho_{D_{2}O}\;=\;3.45\cdot \;10^{-6}A^{-2}$, in SMW $\rho_{SMW}=2.55{\cdot \;10}^{-6}\;A^{-2}$, and in H_2_O $\rho_{H_{2}O}=2.00\cdot 10^{-6}\;A^{-2}.$ The respective SLD profiles of the Native01 and Modified01 SMB are depicted in Figures 3D, 4D. The fit results are reported in Table 2, the corresponding buffer penetration values are given in Supplementary Table 1. We got a convincing fit with a MBP layer with thickness between *d*_MBP,SBL_ = 60–80 Å (the determined parameters are characterized by large error bars, due to the dilute nature of the MBP layer) and a protein concentration in the range of *c*_MBP,SBL_ = 3–7% vol/vol (corresponding to MBP concentrations of around 22 and 51 mg/ml, respectively, when a protein specific volume of 0.73 ml/mg is considered) for both membrane types and both injected MBP concentrations of 0.1 and 1 mg/ml. The MBP layer thicknesses and concentrations as reported in this study are in agreement with previous observations (Krugmann et al., 2020b).

Vesicles were injected onto the MBP/SMB systems and the interaction process was studied with kinetic NR acquisition. The kinetic data will be discussed in the next section, but beforehand we take a look at the steady state specular NR measurement after an almost constant state is reached after 1–2 h. A schematic visualization of that steady-state membrane system in shown in Figure 2C. Experimental NR data of native and EAE modified conditions under steady-state conditions are shown in Figures 3E, 4E including theoretical fits. We can see a strong change in the reflectivity profiles in comparison to the membranes without adhered vesicles. As a fit model we assume a double-bilayer system because the vesicles diffuse to the membrane and adhere to it. In this case, the specular NR profile should be a double bilayer as specular NR only is sensitive for the *z*-structure close to the Si-surface since the penetration depth of the neutrons under such small angle is quite low. However, to account for the part of unfused adsorbed vesicles that extends into the solution, we add in our model a long 800 Å diffuse vesicle layer as last layer with a SLD of 0.7 · 10^−6^Å^−2^ and high roughness and buffer penetration. The thickness of the MBP layer in the double bilayer model has been fitted to *d*_MBP,DBL_ ≈ 30 Å which is close to what we have measured before in between 2 bilayers in small-angle X-ray scattering experiments (Krugmann et al., 2020b). The fitted values of *d*_head_ and *d*_chain_, water penetration and roughness between the layers from the SMB without MBP were again used and constrained in ±5% of the best value around those values. The thicknesses of the second membrane layer (*d*_chain_, *d*_head,inner_, and d_head,outer_) are fixed to the values *d*_chain_ and *d*_head,outer_ of the first layer. They are fixed to *d*_head,outer_ as they are both not attached to the silicon substrate and therefore less ordered. Fitted parameters are the buffer penetration value corresponding to the MBP concentration between the bilayer *c*_MBP,DBL_. The fitted buffer penetration values are given in Supplementary Table 1 and the MBP concentrations in *c*_MBP,DBL_ in Table 2. The SLD-profiles we attain form those fits are shown in Figures 3F, 4F.

The fitted structural models of the three stages of the experiment are summarized visually in Figure 2 including the averaged thickness values of the membrane components. Overall, our characterization by NR of the assembly process under steady-state conditions yields a coherent picture of the assembly process. MBP that is injected as a rather dilute concentration interacts strongly with the first SMB and forms a concentrated fluid phase with concentration in the range of 3–7% and a thickness of 60–80 Å. Upon formation of the second membrane layer the MBP layer thickness is reduced to around half of its initial thickness (~30 Å) and a concentration of 0–1% vol/vol for the 0.1 mg/ml samples and 11% for 1 mg/ml sample. This MBP layer is, hence, regulating the assembly and fusion process of the LUV with the SMB. However, due to the complexity of the system in its final state and the dilute nature of the MBP layer, it appears that our modeling is sensitive only to the thickness of the MBP layer, leading to relative underestimation on protein concentration.

Furthermore, from the buffer penetration value *h*_*chain*,2_ of the second chain section (given in Supplementary Table 1) it is possible to calculate the coverage η of the second bilayer as the hydrophobic chain section is nearly water free in both cases. Therefore, it is possible to calculate the coverage as η = 1 − *h*_*chain*,2_. The definition of the coverage η can be expressed mathematically as the area of the second bilayer *A*_*dbl*_ divided by the total area *A*_*tot*_ of the first bilayer of the sample

(12) $$\begin{array}{l} \eta =\frac{A_{dbl}}{A_{tot}}. \end{array}$$

In Table 2 the coverage values of η are given for the investigated membrane systems.

**Figure 5:**
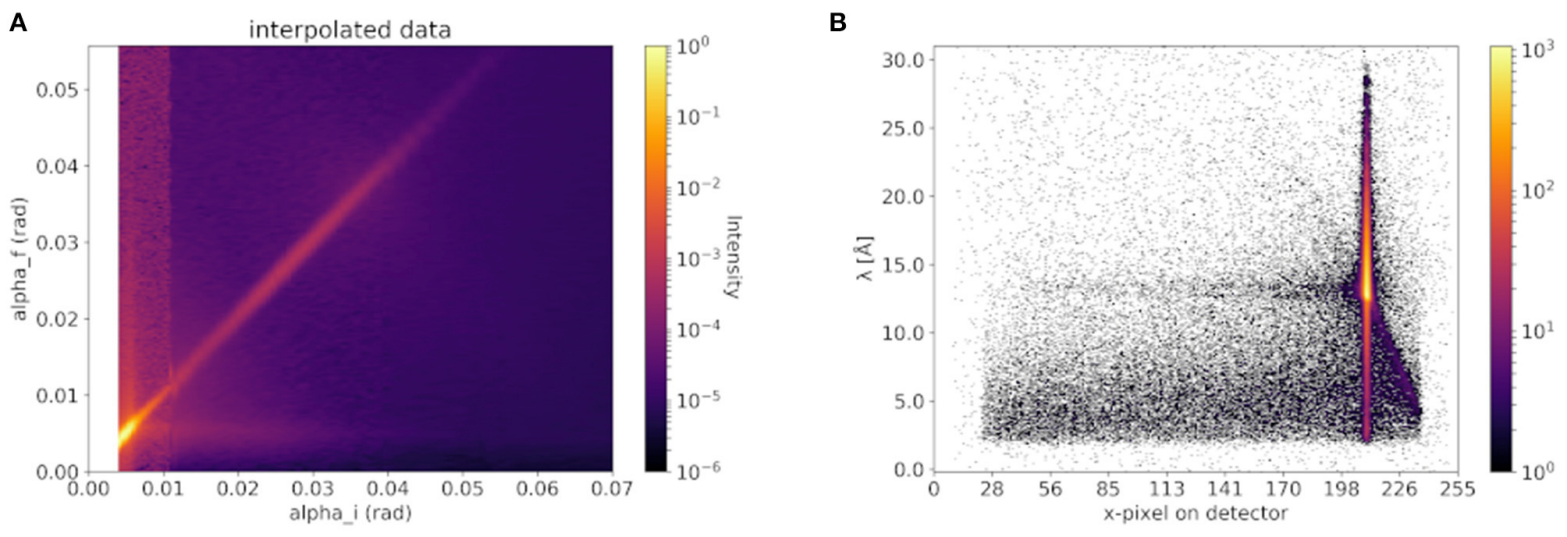
Off-specular signal of vesicle coated membranes seen by **(A)** classical monochromatic and **(B)** TOF neutron reflectometer. In **(A)** an exemplary measurement at the classical monochromatic instrument MARIA (Mattauch et al., 2018) from an EAE-modified sample of our previous paper (Krugmann et al., 2020b) is shown. And in **(B)** an exemplary measurement of an EAE modified membrane at the TOF reflectometry mode using an incident polychromatic neutron beam of the D17 instrument is shown as comparison.

In Figure 5A the 2D off-specular map of an EAE-modified sample in the final steady-state of LUVs bound to the MBP-coated SMB measured at the MARIA reflectometer is given. For technical details of the instrument MARIA, we refer to the instrument paper (Mattauch et al., 2018). In Figure 5B the 2D image of an EAE modified sample of LUVs bound to the MBP-coated SMB measured using the D17 reflectometer is shown. Clearly, an off-specular signal is visible in both cases. This off-specular signal is proof that the samples have strong surface roughness which is an indication for vesicles adhered to its surface (Ott and Kozhevnikov, 2011).

### Kinetics of the Vesicle Adhesion Process

**Figure 6:**
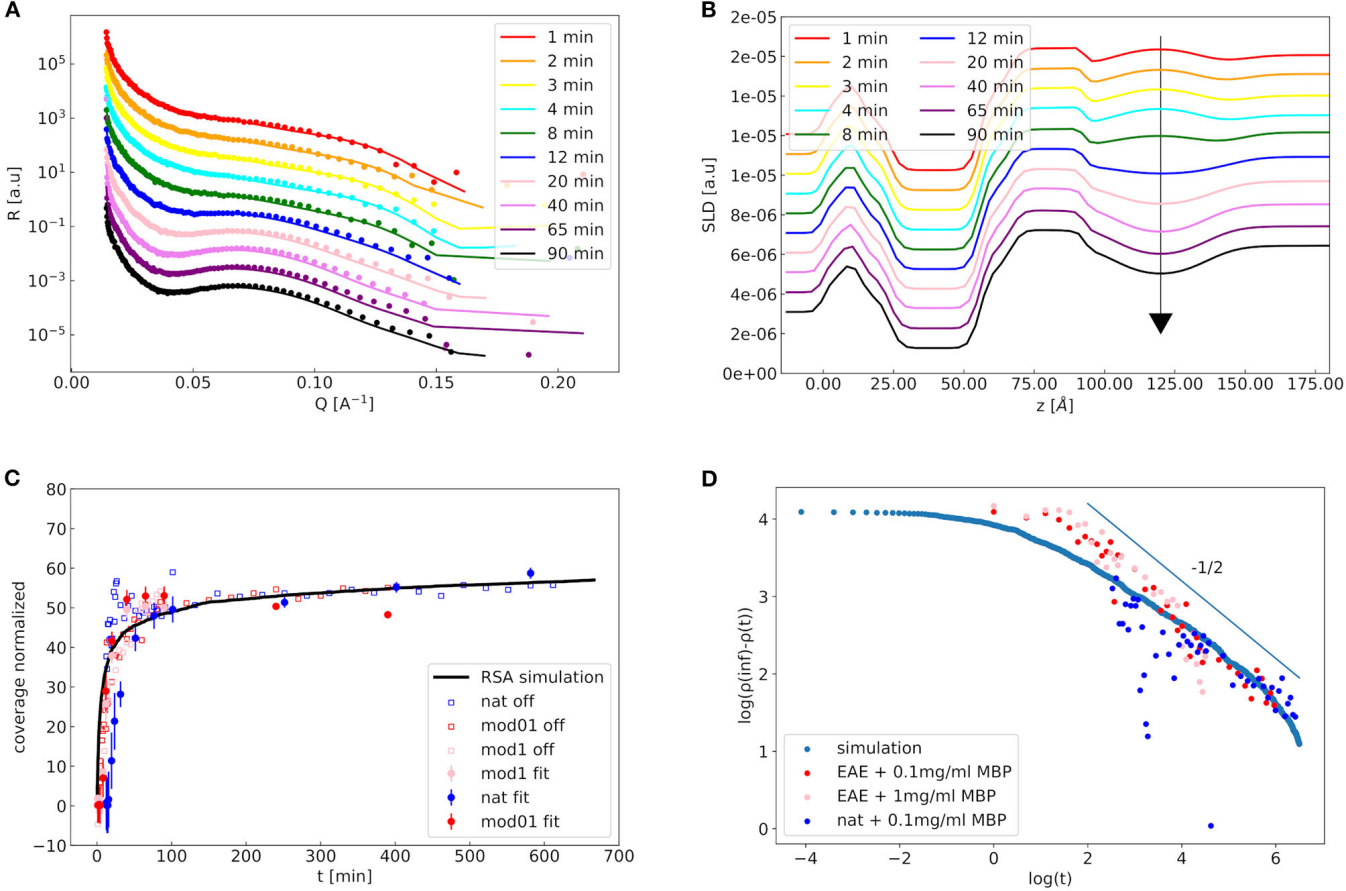
Kinetics of the reflectivity measurements: In **(A)** a waterfall plot of the kinetic reflectivity measurements of the EAE modified membrane with 1 mg/ml MBP is shown (sample Modified1). Experimental NR data are represented by symbols as a function of incubation time. Solid lines are theoretical fits using the double-membrane bilayer model with intramembrane-MBP layer and diffuse vesicle layer for 3D structure of the attached vesicles. Structural parameters of the membrane profile were kept fixed during the individual fits and only the coverage fraction of the second membrane layer (and the diffuse vesicle layer water content and roughness) was fitted. In **(B)** the respective SLD profiles are shown. In **(C)** the kinetics of the off-specular signal of the three membranes (empty symbols), the kinetics of the coverage fit parameter of the three membranes obtained from specular NR data (filled symbols) and an RSA simulation of vesicles with 50 nm radius and 15 nm polydispersity (solid black line) are shown. In **(D)** the log-log-plot of the vesicle coverage ρ(inf)−ρ(*t*) over the time is shown for the off-specular experimental values and the RSA simulation. The thin line indicates power-law behavior with a slope of −1/2, which is the theoretical prediction of an RSA process of flat disks on a surface (Adamczyk et al., 1997).

Time-resolved NR experiments were carried out to understand the process of adhesion. In Figure 6A an exemplary kinetic NR measurement of a modified membrane is shown. To maximize the NR signal and to reduce the incoherent background, time-resolved NR experiments have been performed only at one contrast in 100% D_2_O buffer. Clearly, in the time range of one to a few hours the adhesion process is happening. The off-specular data is growing in this time as well. The specular curves in Figure 6A can be fitted by the earlier explained double bilayer model. Here, only the coverage (η = 1 − *h*_*chain*,2_), water penetration of the diffuse vesicle layer its roughness and the scaling factor are fitted, while the rest of the remaining parameter values are fixed to the ones obtained from the static measurements of the membrane with adhered vesicles. The influence of the head section is not that strong as the contrast to D_2_O is higher to the chain section. In Figure 6B the respective SLD profiles are depicted. In Figure 6C the normalized coverage (scaled to the RSA simulation curve) from the specular fit, the normalized off-specular signal (scaled to the RSA simulation curve with subtracted background) and the behavior represented by the RSA simulation curve are plotted together. The important observation here is that the normalized experimental coverage values obtained from both specular and off-specular NR data are identical within the statistical uncertainty and their time-dependence can be reproduced by the theoretical model of an RSA simulation.

RSA is a model in which particles diffuse and adhere to a surface and cannot detach from it. The details of the RSA simulation are explained in the Materials and Methods section. Strikingly, the kinetics of the specular data—representing the formation of a double bilayer—and the off-specular data—representing the adhesion of vesicles to the surface—follow the same trend and can both be described by the same RSA simulation. We can conclude here that both signals are governed by the same kinetic time constant of the diffusion driven adhesion of the vesicles. The deformation of the vesicles which is responsible for the double bilayer formation is, therefore, quasi-instantaneous in comparison to the adhesion process. In RSA the vesicles that adsorb to the membrane are described as disks that—when adsorbed—exclude a certain area *A*_*ex*_. After waiting for an infinite amount of time (*t* = inf) the coverage of vesicles ρ(*t*)–which is different from η as not the whole excluded diameter is in contact with the membrane—a coverage ρ(inf) is reached. To verify if RSA is the correct kinetic process to describe our data we plot the logarithm of ρ(inf)-ρ(t) over the logarithm of time in Figure 6D. This should give a slope of −1/2 for an RSA process of disks on a surface, which is indeed the case of our NR data when we exclude the first initial time points (Hinrichsen et al., 1986).

#### Calculation of Adhesion Energy per MBP

From the above-mentioned results, it is possible to estimate the adhesion energy per area *k*_*a*_. This is achieved by minimizing the free energy which is constituted of the bending energy and the adhesion energy (see section Free Energy of a Vesicle). By accounting for some geometrical assumptions (see section Combining RSA simulations and neutron reflectivity measurements to access α_f_) we can estimate the adhesion energy per area *k*_*a*_ of the native and modified membranes with 0.1 mg/ml MBP. Values of *k*_*a*_ are given in Table 3. With this we can calculate the interaction energy per MBP molecule ϵ_*MBP*_.

(13) $$\begin{array}{l} \epsilon_{MBP}=k_{a}\cdot A_{MBP}, \end{array}$$

where *A*_*MBP*_ is the area per MBP molecule we take from Krugmann et al. (2020b) calculated with the MBP molecule volume from Stadler et al. (2014). Input values of *A*_*MBP*_ and the obtained values of ϵ_*MBP*_ are compiled in Table 3 for the native and EAE-diseased membranes. We can see that the so-estimated adhesion energy per MBP-molecule in native membranes—calculated by this model—is larger as in EAE modified membranes which might explain the formation of a stable myelin sheath. The *A*_*MBP*_ values calculated from fits of D17 data that are presented in this publication have large statistical uncertainty, but they are in agreement with values of *A*_*MBP*_ that we have reported previously (Krugmann et al., 2020b). The large error bars of the D17 fit parameters *A*_*MBP*_ (Table 3) lead to high errors of ϵ_*MBP*_ as well. Therefore, it is not possible to draw any conclusions on the difference between native and EAE-diseased membranes using these values.

In the case of the EAE modified membrane with 1 mg/ml MBP concentration, we have a coverage fraction η_*dbl*_ of around 39%. This would lead to a high value of *k*_*a*_ in the range of the value of silicon $k_{a}\left(Si\right)=0.5-1\;mJ/m^{2}$ where we already observe vesicle fusion triggered by the strong adhesion (Anderson et al., 2009). Therefore, we believe that it is unphysical and probably we have partial vesicle fusion occurring. This case is not covered in our model and would of course increase η_*dbl*_ without the vesicles needing to be flattened as much. Therefore, the calculated *k*_*a*_ would decrease dramatically. Partial vesicle fusion might also happen in the lower protein concentration case where the values are close to those measured in silica. In that case our calculated values of *k*_*a*_ and ϵ_*MBP*_ might be overestimated.

## Conclusions

In this manuscript, we report on the adhesion mechanisms of LUV onto a supported membrane that was coated with an MBP layer. Structural characterization at the different stages of membrane assembly process has been performed under steady-state conditions, while the vesicle fusion process has been monitored as function of the incubation time. The overall aim was to investigate differences between native and EAE modified biomimetic cytoplasmic myelin membranes and to gain information on the myelin sheath assembly process at a molecular scale. From the steady-state experiments we demonstrate that a concentrated MBP layer is formed on top of a single SMB with a protein concentration of around 3–7%. Adhesion of LUV with local formation of a second membrane bilayer results in an MBP layer that is reduced in thickness. Concerning the LUV adhesion kinetics, we show that the adhesion of the vesicles can be modeled by an RSA simulation. In addition, we provide evidence that the vesicles are reshaping after the adhesion. Their contact surface with the membrane is maximized until the elasticity of the membrane leads to a force in opposite direction and an equilibrium state is established. Our measurements show that this process depends on both membrane type and MBP concentration, while its kinetics is entirely limited by the diffusion of the vesicles toward the membrane surface agreeing with the observed RSA mechanism. In combination with the bending rigidity of those membranes, which we investigated in earlier studies (Krugmann et al., 2020b), we can provide an estimate of the interaction force per MBP molecule: In the native-like membrane case it is larger than in the EAE-diseased membranes. Our new results can also help with the interpretation of our last paper (Krugmann et al., 2020b). In that study, we have seen that LUV having EAE modified lipid composition tend to stick to each other by a strong attractive interaction being mediated by the binding strength of MBP. As we show in here, this seems not to be because of a stronger interaction of MBP with these EAE-diseased membranes as the adhesion energy per MBP-molecule is higher for native membrane than for the EAE-diseased membrane. Instead, the driving force of that effect appears to be due to the higher MBP concentration in the MBP layer on top of EAE modified membranes as compared to that of native-like membranes.

We found indications that partial vesicle fusion is occurring when EAE membranes were treated with the higher MBP concentration of 1 mg/ml. By applying external stimuli like osmotic shock or temperature shocks it might be possible to achieve complete vesicle fusion resulting in a perfect double bilayer system with an intercalated MBP layer in between the two bilayers. This would be a significantly improved model system to study the initial growth process of the myelin sheath from a molecular perspective, and it would allow us to study basic biophysical properties of artificial biomimetic nerve cells *in vitro*.

## Data Availability Statement

The datasets analyzed for this study can be found in the ILL data repository (data.ill.eu) (Krugmann et al., 2020a).

## Author Contributions

AS, AR, AK, and SF designed the research. BK, AK, LH, and SM performed the research. BK and AK analyzed the data. DL contributed analytical modeling. BK and AS wrote the manuscript with input from all other authors. All authors read and approved the finalized manuscript.

## Conflict of Interest

The authors declare that the research was conducted in the absence of any commercial or financial relationships that could be construed as a potential conflict of interest.

## Footnotes

^1^https://avantilipids.com/ (accessed March 1, 2021).

^2^*Biomolecular Scattering Length Density Calculator*. http://psldc.isis.rl.ac.uk/Psldc (accessed March 17, 2021).
