# Supplementary material for: Adhesion Process of Biomimetic Myelin Membranes Triggered by Myelin Basic Protein
Source: Front Chem. 2021 May 4;9:631277. doi: 10.3389/fchem.2021.631277 (PMC8129001; doi:10.3389/fchem.2021.631277)
Supplement: Supplementary file 1 [file Data_Sheet_1.PDF]

Supplementary Material

Adhesion process of biomimetic myelin membranes triggered by myelin basic protein

Benjamin Krugmann[1,3], Alexandros Koutsioubas[1], Luman Haris[2,3], Samantha Micciulla[4], Didier Lairez[5], Aurel Radulescu[1], Stephan Förster[2], Andreas M. Stadler[2,3,*]

[1]Jülich Centre for Neutron Science at MLZ, Forschungszentrum Jülich GmbH, Lichtenbergstr. 1, 85748 Garching, Germany

[2]Jülich Centre for Neutron Science (JCNS-1) and Institute for Biological Information Processing (IBI-8), Forschungszentrum Jülich GmbH, 52425 Jülich, Germany

[3]RWTH Aachen University, Institute of Physical Chemistry, Landoltweg 2, 52056 Aachen, Germany

[4]Institut Laue-Langevin, 71 Avenue des Martyrs, 38042 Grenoble Cedex 9, France

[5]Laboratoire des Solides Irradiés, École polytechnique, CEA, CNRS, Institut Polytechnique de Paris 91128 Palaiseau cedex, France

*** Correspondence:**
Andreas M. Stadler
a.stadler@fz-juelich.de

1 Supplementary Figures and Tables

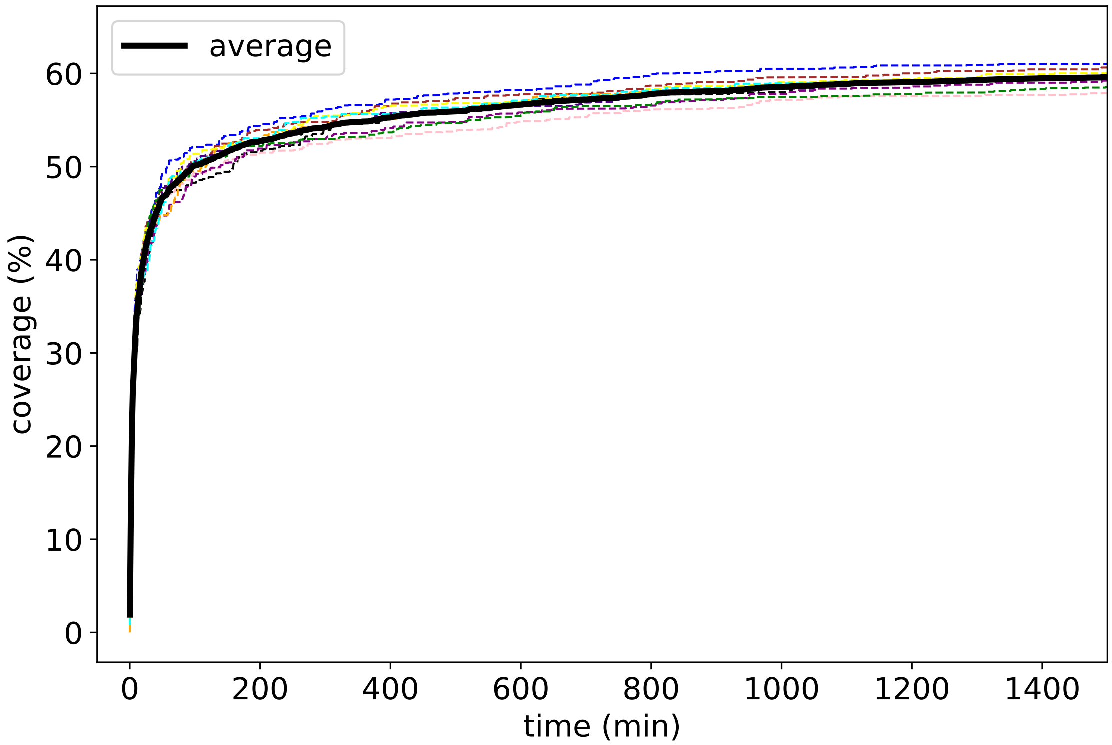

Supplementary Figure S1. Simulation of the maximum coverage of the membranes with 0.3 polydispersity. Here, the timestep 10 s was used and the simulation was repeated 10 times.

Supplementary Table S1. Buffer penetration values of the different membrane systems in %. Native membrane (N), EAE-diseased membrane (D). Typical absolute errors are: head sections +/- 10 %, chain sections +/- 1-3 %, MBP +/- 2 %.

system	$head_{i1}$	$chain_1$	$head_{o1}$	MBP	$head_{i2}$	$chain_2$	$head_{o2}$
N	16	0	48	--------	--------	--------	--------
N + 0.1 mg/ml MBP	16	5	50	97	--------	--------	--------
N + 0.1 mg/ml MBP + LUV	16	5	49	99	75	74	78
D	22	1	54	--------	--------	--------	--------
D + 0.1 mg/ml MBP	21	5	57	95	--------	--------	--------
D + 0.1 mg/ml MBP + LUV	23	5	55	100	81	66	79
D	36	0	50	--------	--------	--------	--------
D + 1 mg/ml MBP	35	5	51	93	--------	--------	--------
D + 1 mg/ml MBP + LUV	35	5	50	89	78	61	72

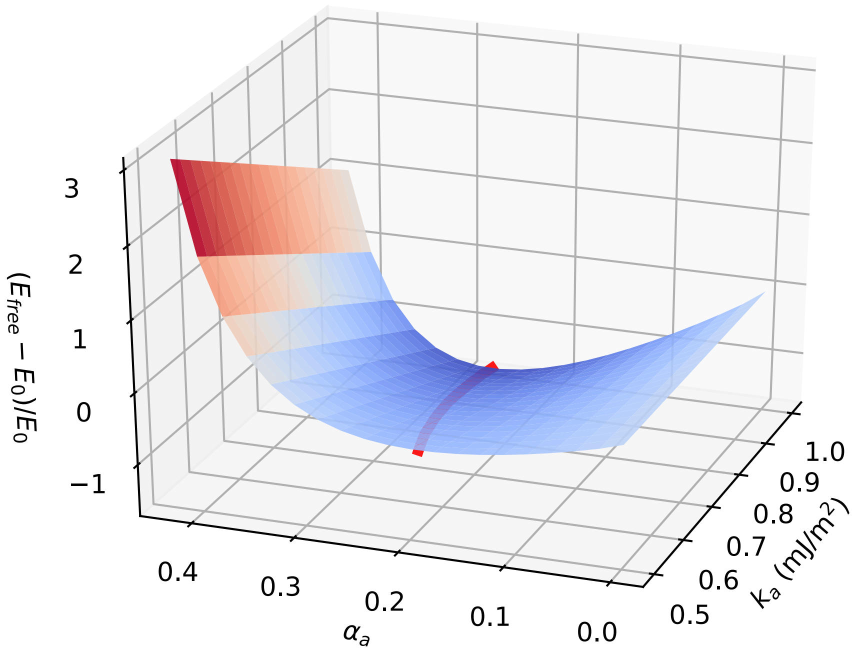

Supplementary Figure S2. Variation of the free energy E_{free} of an adsorbed vesicle with the adhesion constant k_a and the adhesive-area fraction α_f. E_0 is the free energy of the spherical vesicle. Calculations are done for vesicle radius *R* = 50 nm and bending elastic modulus $k_c = 1.37 \cdot 10^{-19}\ J$. The red line corresponds to the minimum for E_{free}

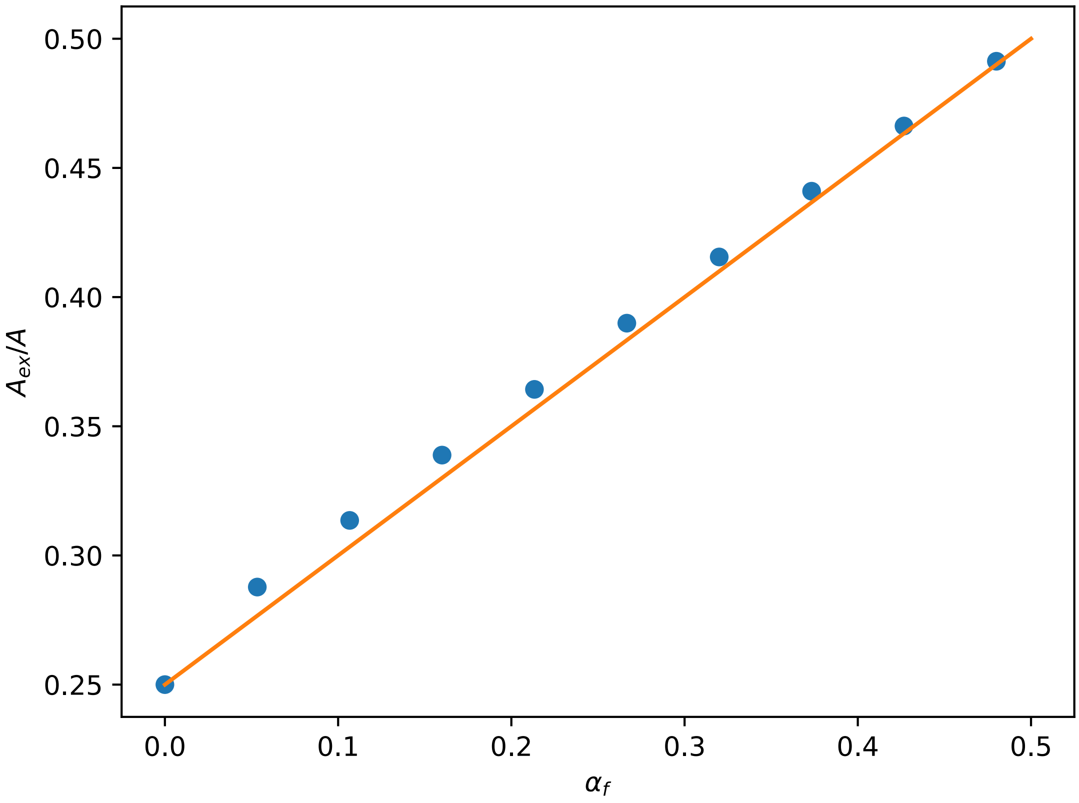

Supplementary Figure S3. Dependency of A_{ex}/A over α_f computed by the least-energy surface. The points show an almost linear behavior.
